# Simultaneous treatment of pterygium complicated with conjunctivochalasis: analysis of pterygium excision and conjunctival autotransplantation combined with sclera fixation

**DOI:** 10.1186/s12886-015-0057-4

**Published:** 2015-08-12

**Authors:** Xiao-Yi Yu, Zhan-Yu Jian, Wei Wu, Xiao-He Lu

**Affiliations:** Department of Ophthalmology, the first affiliated hospital of Guangzhou University of Chinese Medicine, Guangzhou, Guangdong Province 510405 China; Department of Ophthalmology, Zhujiang hospital of southern medical university, Guangzhou, Guangdong Province 510282 China

**Keywords:** Pterygium, Conjunctivochalasis, Simultaneous treatment, Pterygium excision, Conjunctival autotransplantation, Sclera fixation, Therapeutic contact lens

## Abstract

**Background:**

This prospective study investigated the safety and efficacy of a therapeutic method of treating pterygium complicated with conjunctivochalasis, using pterygium excision and conjunctival autotransplantation combined with sclera fixation, followed by therapeutic contact lens application.

**Methods:**

Fifty-seven patients (83 eyes) diagnosed as pterygium complicated with conjunctivochalasis, at our hospital from July 2011 to June 2012, were selected. Patients were treated with pterygium excision and conjunctival autotransplantation combined with sclera fixation surgery, then therapeutic bandage contact lenses were applied. The efficacy of simultaneous surgery was evaluated based on vision changes, tear dynamics, and other complications. Histopathological changes were investigated on removed bulbar conjunctival tissue, using hematoxylin eosin (HE) and Masson’s trichrome staining.

**Results:**

(1) Three months after the operation, the success of simultaneous surgery in the treatment of pterygium was 97.6 %, and the recurrence was 2.4 %. Based on subjective evaluation, the success of the simultaneous treatment of conjunctivochalasis was 95.2 %, and failure was 4.8 %. Based on objective evaluation, the success rate was 94.0 % and the recurrence rate was 6.0 %. (2) Visual acuity of the 83 eyes was significantly improved after surgery, and was statistically significant (*X*_2_ = 10.29, *P* < 0.05). (3) Three months after surgery, the height and integrity of the tear meniscus, tear film break-up time, and chloramphenicol test results of the 83 eyes were significantly improved and there was a statistically significant difference (*X*_2_ the height and integrity of tear meniscus = 147.24, *X*_2_ tear film break-up time = 81.17, *X*_2_ chloramphenicol test = 17.41, *P* < 0.01). (4) Complications after the operation such as granulation hyperplasia, constrictive fornix, oculomotor defect, and other complications were not observed. (5) Pathological observations, using HE and Masson’s trichrome staining of removed bulbar conjunctival tissue, showed several pathological changes, including obvious squamous epithelial hyperplasia, parakeratosis, basal cell pigmentation, lamina propria hemorrhage, infiltration of lymphocytes, and reduction of elastic fibers and collagen fibers.

**Conclusion:**

Pterygium excision and conjunctival autotransplantation, combined with sclera fixation followed by therapeutic contact lens use was safe, effective, and suitable for simultaneous treatment of pterygium complicated with conjunctivochalasis.

## Background

Pterygium is a common clinical ocular surface disease, mainly characterized by fibrovascular overgrowth of bulbar conjunctiva across the limbus onto the cornea, thus affecting appearance, vision and comfort [1]. Now it was believed that its formation and recurrence were caused by ultraviolet light, dust, dryness, heat or other environmental factors [2]. Conjunctivochalasis as a kind of ocular disease resulted from redundance of conjunctiva epithelium or stroma in the lower forniceal conjunctiva or the lower half of the bulbar conjunctiva, which caused the dysfunction of tears over ocular surface [3]. Pterygium complicated with conjunctivochalasis was common ocular surface diseases, which leaded to ocular irritation symptoms such as dryness and tears [4]. And these diseases would affect appearance, or even vision, work and life seriously.

In this study, pterygium excision and conjunctival autotransplantation combined with sclera fixation followed by therapeutic contact lens was applied to simultaneously treat pterygium complicated with conjunctivochalasis. The redundancy of inferior bulbar conjunctiva in the patients with conjunctivochalasis was utilized as autograft to repair sclera wound caused by pterygium excision. This method resumed the normal surface of eye conjunctiva and promoted recovery of the morphology and function of conjunctiva. Then vision changes, tear dynamics and other complications were evaluated. Meanwhile, the removed part of bulbar conjunctival tissue was investigated based on Hematoxylin Eosin (HE) staining and Masson Trichrome, which showed that the pathological changes in conjunctival tissues included the obvious squamous epithelial hyperplasia, parakeratosis, basal cell pigmentation, lamina propria hemorrhage, infiltration of lymphocytes, elastic fibers and reduction of collagen fibers. To our best knowledge, although some special cases involved in simultaneous pterygium and chalasis surgery has been reported by another group previously in 2013 [5], it was the first time to report comprehensive evaluation of simultaneous treatment of pterygium complicated with conjunctivochalasis. This study aimed to provide the detail of this effective method and confirmed that it was safe, effective, and suitable for simultaneous treatment of pterygium complicated with conjunctivochalasis. Now the report was as follows.

## Methods

The study was approved by the Hospital Scientific Committee “*Committee of Zhujiang Hospital of Southern Medical University*” and written informed consent was obtained from all patients included in the study. 57 patients (83 eyes) diagnosed as pterygium complicated with conjunctivochalasis in our hospital from July, 2011 to June, 2012 were selected with an average of 70.6 ± 6.3 years old. All 83 eyes were primary nasal pterygium complicated with different degrees of conjunctivochalasis, in which the head of pterygium had grown into limbus corneae more than 2 mm. Conjunctivochalasis were divided into different stages according to the theory of Zhang *et al.* [6] (15 eyes with Grade-I (18.1 %), 41 eyes with grade-II (49.4 %), 23 eyes with grade- III level (27.7 %) and 4 eyes with grade- IV (4.8 %)). Exclusion criteria: patients with systemic or other partial ocular diseases such as hyperthyroidism, acute ocular inflammation, xerophthalmia, lacrimal disease, high tension of lower margo palpebrae, entropion and other ocular surface diseases.

1. 4 g/L Hydrochloric Acid Oxybuprocaine Drops and 20 g/L Lidocaine (0.5 ml) were used for topical anesthesia and pterygium anesthesia respectively. (2) Under surgical microscope, the conjunctival tissue on both sides of the neck of pterygium was cut off, and the surface of conjunctiva on the neck of pterygium was dissected parallel to limbus corneae. The conjunctiva was severed from the underlying fibrous tissue till plica semilunaris. Then the pterygium at the point of plica semilunari was excised, and the head of pterygium was avulsed reversely from the point of plica semilunari to the center of the cornea. After that, the blade was applied to scrape the abnormal tissue remnant, which ensured excellent polishing and smoothing of the surface. Finally, hemostasis was achieved with coagulation of the bare sclera. (3) The width at the limbus, width of the nasal lesion and distance from the limbus to the margin to be excised would be measured to determine the size of conjunctival autograft. Then a piece of conjunctival flap with limbal stem cells harvested in inferior corneal limbus was transferred to the bare sclera after the pterygium excision. And the reflected conjunctival flap was pressed down onto the corneal, and the subepithelial fibers should be made sure to overlay the sclera taut. Additionally, its limbus should be aligned with the limbus of bare scleral bed. The graft was then attached to the perilimibal conjunctiva and episclera with 10–0 nylon sutures in simple interrupted fashion. On the other hand, the excess bulbar conjunctiva was removed according to the relaxation degree of conjunctiva, and the bulbar conjunctiva was sutured to inferior limbus corneae with 10–0 nylon sutures in simple interrupted fashion. The incision on the nasal and temporal site was sutured in continuous manner to the episclera. At the same time, the bulbar conjunctiva was sutured to episclera at 6–8 cm below limbus corneae with 10–0 nylon sutures in simple interrupted fashion. When suturing under the microscope, the angle and depth of needle insertion should be controlled to avoid puncture of ocular wall. (4) After the operation, eyes were treated with Tobramycin and Dexamethasone Ophthalmic Ointment, and patients were proposed to wear therapeutic corneal bandage lens. All patients underwent a simultaneous combined surgery; monocular operation cost 30–40 min with no complaint of pain, less bleeding and without abnormal complications.

From the first day after operation, Tobramycin and Dexamethasone Ophthalmic Suspension Eye Drops were treated 5 times per day, Pranoprofen Eye Drops were treated 3 times per day, Sodium Hyaluronate Eye Drops were treated 3 times per day, and FGF Eye Gel Eye Drops were treated 3 times per day. Taper the use of eye drops according to the healing degree of conjunctival graft, corneal and conjunctiva, and stop using them in 2–3 months. The 10–0 nylon suture was removed 10 days after surgery and the corneal bandage lens were removed 20 days after surgery. The removed part of the bulbar conjunctival tissue was investigated to observe its histopathological changes based on Hematoxylin Eosin (HE) staining and Masson trichrome.

Patients would be paid a visit at first week, first month, and third month respectively after the operation. The discomforts would be asked such as eye irritation, foreign body sensation and epiphora. The complications would be recorded such as conjunctival fornix shallow and eye movement disorders, the naked visual acuity, conjunctival flap, corneal condition, height and integrity of Tear Meniscus, Tear Film Break-up Time, Chloramphenicol Test and other circumstances of the patients, which would be compared with pre-operation followed by making curative effect judgment.

SPSS16.0 statistical software was applied for statistical analysis, two samples were compared by using *t* test, and *X*_2_ test was used to compare rates.

## Results and discussion

**Fig. 1:**
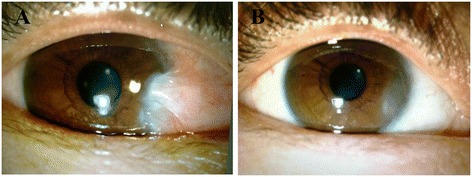
**a** Eye of patients before operation; **b** Eye of patients one month after operation

Criteria for pterygium: (1) Healed: free of pterygium recurrence and free of conjunctival congestion; (2) Effectiveness: free of pterygium recurrence, but with conjunctival congestion; (3) Ineffectiveness: pterygium recurrence. Three months after operation, among the 83 eyes, 64 eyes were healed (77.1 %), 17 eyes were effective (20.5 %), 2 eyes were ineffective (2.4 %) and the total success rate was 97.6 % (see Table 1, Fig. 1).

**Table 1:** Three months later, the analysis of the simultaneous treatment on the patients of pterygium complicated with conjunctivochalasis

	Evaluative criteria	Recovery (n (%))	Effectiveness (n (%))	Ineffectiveness (n (%))
pterygium	objective	64 (77.1)	17 (20.5)	2 (2.4)
Conjunctivochalasis	subjective	58 (69.9)	21 (25.3)	4 (4.8)
	objective	62 (74.7)	16 (19.3)	5 (6.0)

*n (%)* the number and percentage of eyes

Criteria for conjunctivochalasis: (1) Subjective evaluation on curative effect: (i) Recovery: the symptoms disappeared completely; (ii) Effectiveness: with no obvious symptoms; (iii) Ineffectiveness: there was no improvement. (2) Objective evaluation on curative effect: (i) Recovery: there was no conjunctiva congestion in the surgical area, and the conjunctiva attached to the scleral surface; (ii) Effective: there was conjunctival nodular hyperemia and scarring in surgical area; (iii) Recurrence: conjunctiva in the surgical area was still loose and wrinkled. 3 months after operation, among the 83 eyes, (1) according to subjective evaluation on curative effect, 58 eyes were recovered (69.9 %), 21 eyes were effective (25.3 %), 4 eyes were ineffective (4.8 %) and the total success rate was 95.2 %; (3) according to objective evaluation on curative effect, 62 eyes were recovered (74.7 %), 16 eyes were effective (19.3 %), 5 eyes were ineffective (6.0 %) and the total success rate was 94.0 % (see Table 1, Fig. 1). Recurrent cases were all at I-II level of conjunctivochalasis, which mainly featured conjunctivochalasis accumulation in the media and lateral angle of eyes.

Criteria of the vision changes: an international visual acuity chart was applied to compare the visual acuity of patients before surgery with that in 3 months after surgery. That improved more than 2 lines was considered as vision improvement, that decreased more than 2 lines was considered as visual loss and that fluctuating less than 2 lines was considered as not significant; The visual acuity of patients before operation ranged from 0.06 to 0.8, the median was 0.3; 3 months after surgery, the vision ranged from 0.08 to 1, the median was 0.5. Three months after operation, the visual acuity of 56 eyes was improved (67.5 %), and the visual acuity of 27 eyes was not significant changed (32.5 %). The difference was statistically significant (*X*_2_ = 10.29, P <0.05, see Table 2).

**Table 2:** Vision changes of the patients before operation and 3 months after operation

	≤0.1	0.2 ~ 0.3	0.4 ~ 0.5	0.6 ~ 0.7	≥0.8
Before operation	6	14	33	22	8
3 months after operation	2	5	29	33	14

By *X*_2_ test, the difference was statistically significant (*X*_2_ = 10.29, *P* <0.05)

Criteria of the dynamic changes of tear fluid over ocular surface: (1) the height and integrity of the tear meniscus: (i) Normal: more than 0.3 mm; (ii) Abnormal: less than 0.3 or tear meniscus appeared irregular, incomplete or interrupted. (2) Tear film break-up time (BUT): (i) Normal: more than 10s; (ii) Abnormal: less than 10s. (3) Chloramphenicol Test: (i) Normal: less than 10 min; (ii) Abnormal: more than 10 min. 3 months after surgery, the height and integrity of Tear Meniscus (*X*_2_ = 147.24, P <0.01), Tear Film Break-up Time (*X*_2_ = 81.17, P <0.01), Chloramphenicol Test (*X*_2_ = 81.17, P <0.01) were investigated, the difference was statistically significant (see Table 3).

**Table 3:** Ocular dynamics changes of the patients before operation and 3 months after operation

	TM (n (%))	TFBT (n (%))	CT (n (%))
Before operation	0 (0.0 %)	14 (16.9 %)	22 (26.5 %)
3 months after operation	78 (94.0 %)	72 (86.7 %)	56 (67.5 %)
*X*_2_	147.24	81.17	17.41
P	<0.01	<0.01	<0.01

(1) *n (%)* the number and percentage of normal eyes; (2) *TM* Tear Meniscus; (3) *TFBR* Tear Film Breakup time; (4) *CT* Chloramphenicol Test

(1) All patients have mild corneal irritation symptoms before taking out stitches. And the symptoms would ease following taking out stitches in 10th day after operation. (2) And 20th day after operation, the corneal bandage lens would be taken out and the symptoms would disappear basically. (3) Graft would have different degrees of edema within 3 days, which subsequently eased gradually. Grafts would survive after 3 months and fused with surrounding conjunctival tissues. (4) Three days after operation, the dehiscence in nasal conjunctival wound was observed in one eye with unknown reasons. After one stitch suture with 10–0 nylon, it recovered smoothly. (5) There were no patients with granulation hyperplasia, subfornical constrictive or other abnormal symptoms.

**Fig. 2:**
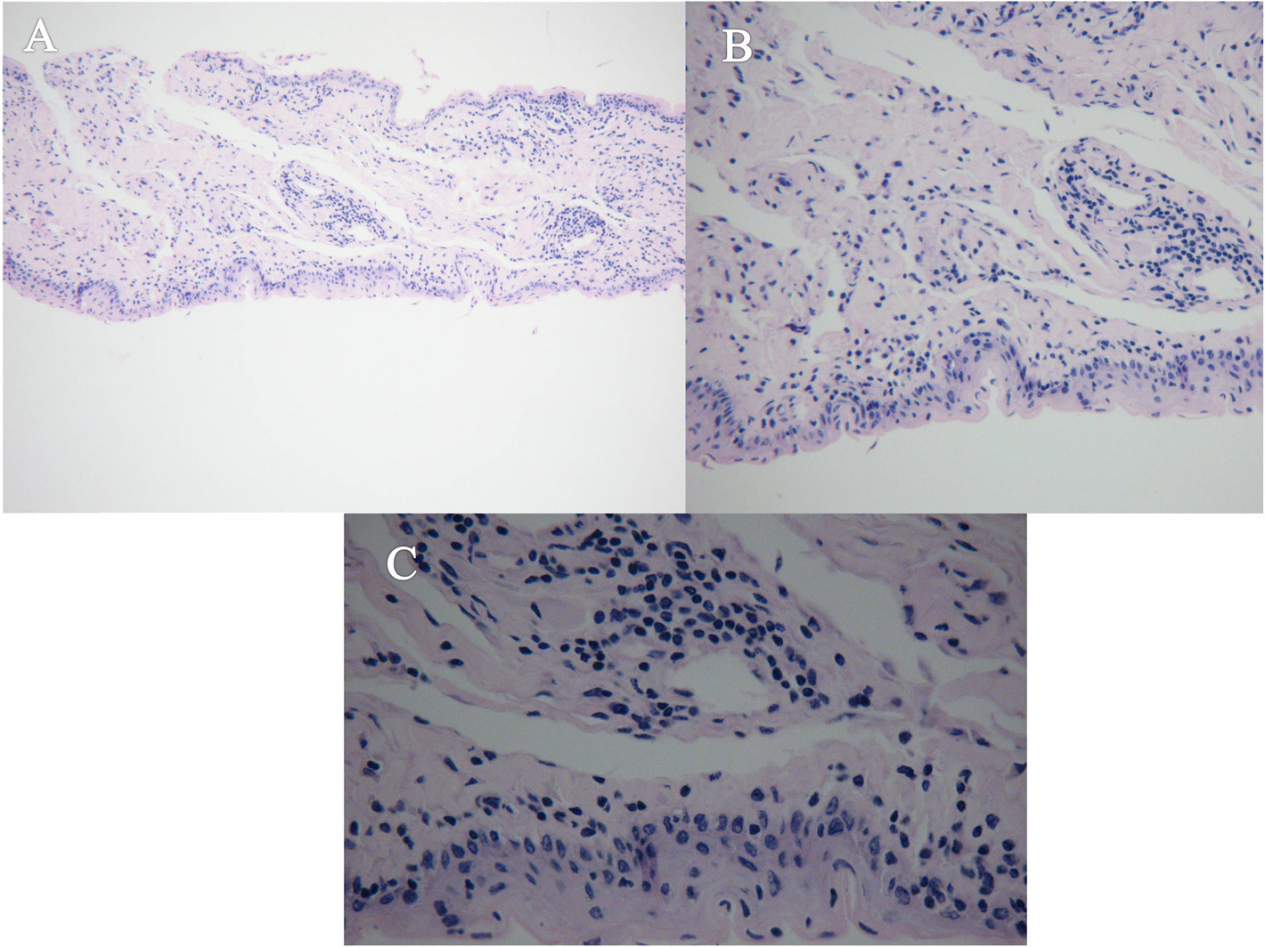
The results of Hematoxylin Eosin (HE) staining were showed as follows; **a** HE X 100; **b** HE X 200; **c** HE X 400

**Fig. 3:**
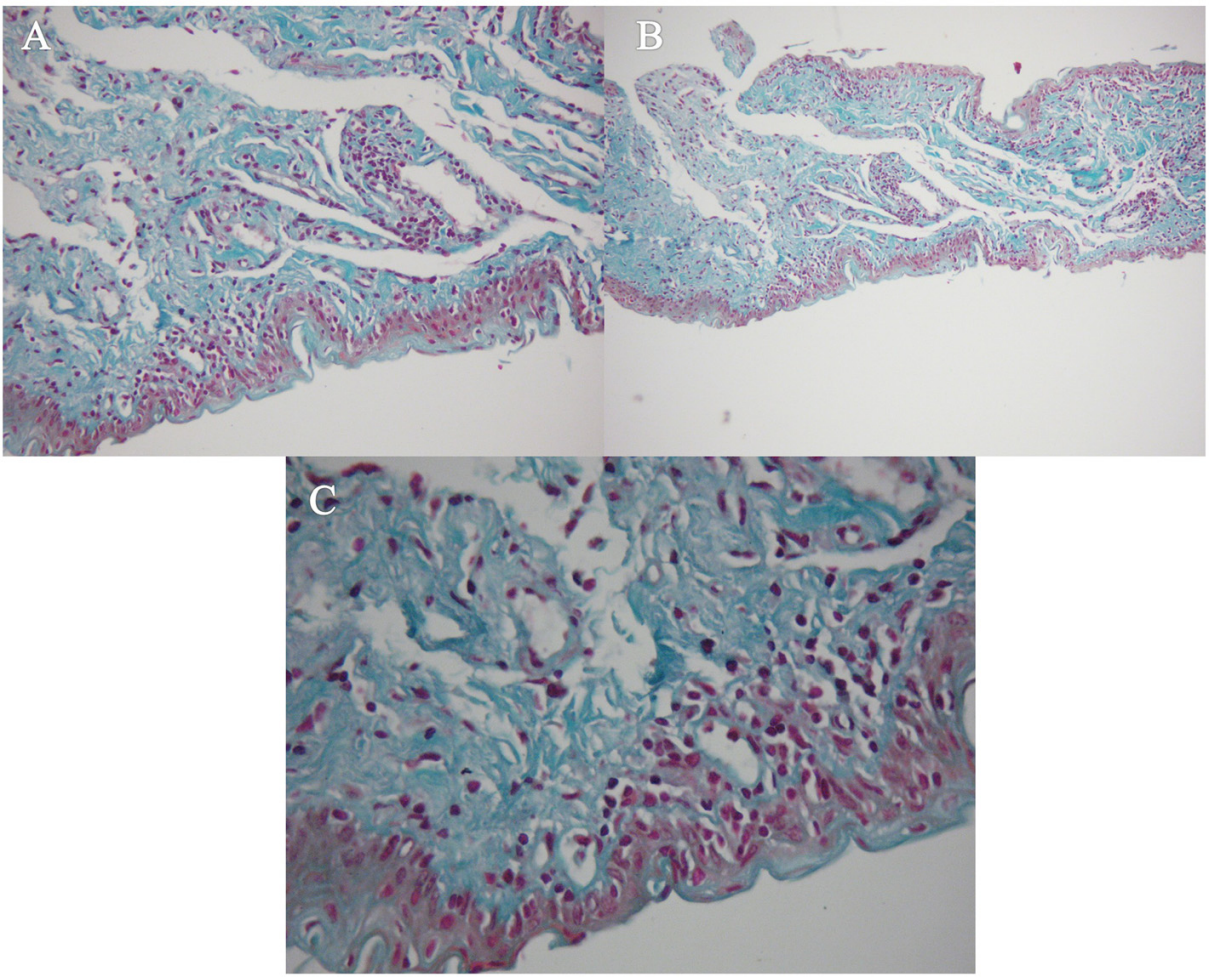
The results of Masson Trichrome were showed as follows; **a** Masson X 100; **b** Masson X 200; **c** Masson X 400

Pathological changes: HE stains and Masson trichrome staining showed that the pathological changed in removed conjunctival tissues including the obvious squamous epithelial hyperplasia, parakeratosis, basal cell pigmentation, lamina propria hemorrhage, infiltration of lymphocytes, elastic fibers and reduction of collagen fibers (see Figs. 2 and 3).

Pterydium and conjunctivochalasis were common clinical ocular surface diseases. There were various methods of pterygium surgery [7]; Mahdy and others believed that pterygium excision combined with limbal stem cell transplantation was the best method with the lowest recurrence rate [8]. In this method, limbal stem cell was mainly obtained from upper temple. In spite of the operation was simple, it might cause some complications of upper conjunctiva including the formation of subconjunctival scarring and the stenosis, which caused unnecessary trouble for the possible surgeries in the future, such as cataract operation and anti-glaucoma operation [9]. Patients with Grade II-IV conjunctivochalasis, who were clinically ineffective to drug therapy, needed treatment of surgery. The main principle was to remove the excessive conjunctival tissue, reconstruct the ocular surface, improve symptoms and appearance, and prevent the recurrence [6, 10].

Generally, when treated of single pterygium, the superior conjunctiva was used to perform pterygium surgery with conjunctival autografting. However, as for pterygium complicated with conjunctivochalasis, the excessive conjunctiva occurred usually inferiorly. Therefore, it was better to excise inferior conjunctival tissue with limbal stem cells. Amniotic membrane was the innermost layer of the fetal membranes and the amniotic membrane transplantation (AMT) was reported as allograft to treat pterygium [11]. Our research applied simultaneous surgery involved in autologous transplantation, which could avoid several complications caused by allograft, such as inflammatory response and rejection. Additionally, conjunctival autotransplantation could relief the flabby and accumulation parts of inferior conjunctiva, and provided sufficient limbal stem cells and conjunctival flap to promote the healing of corneal epithelium, inhibit the growth of fibrous tissue, reestablish barrier function of limbus cornea and reduce relapse rate of pterygium. And recent research showed that both pterygium and conjunctivochalasis were associated with the decrease of mucoprotein [12]. The autotransplantation of inferior bulbar conjunctiva to sclera bare area on nasal side was for minimal excision of conjunctival tissues to save conjunctival goblet cells as much as possible, which could prevent further decrease of mucoprotein in tear fluid. The utility of conjunctival autograft to repair the sclera could resume the normal surface of eye conjunctiva and promote recovery of the morphology and function of conjunctiva [8, 13]. We needed to decide the amount of excision and morphology according to the degree of conjunctivochalasis, and trim and suture according to the required size of conjunctival graft. We found that the removed conjunctiva from patients with Grade III-IV conjunctivochalasis was enough to be used as conjunctival graft to repair the scleral wound. In case that conjunctival graft from patients with Grade I-II conjunctivochalasis was too narrow, more tension suture could be considered.

Anti FGF topical medications was mainly applied for invasive and recurrent pterygium, which should be limitedly applied for treating pterygium in clinical operation, especially for our simultaneous treatment of pterygium complicated with conjunctivochalasis. The reasons were as follows: (1) Although Mitomycin C (MCC) was applied as a kind of anti FGF topical medications for pterygium excision [14], it was still showed that there was no significant difference between these patients with or without the intraoperative application of Mitomycin C; (2) Furthermore, some complications including irritation, lacrimation and photophobia were more common when simple excision was followed by MMC [15]. (3) In addition, the operative treatment of conjunctivochalasis needed certain fibrous tissue proliferation to form scarring and prevent recurrence. Therefore, the simultaneous surgery of patients with pterygium and conjunctivochalasis should avoid the application of anti FGF topical medications.

In addition, as *Hu CX et al.* [16] reported in 2006, the total success of crescent shaped conjunctiva resection combined with scleral fixation was 91.48 %, which was higher than the respective success of single crescent shaped conjunctiva resection (87.5 %) or conjunctival suture fixation (75.0 %). In this research, we adopted the method of crescent shaped conjunctiva resection combined with scleral fixation. And 3 months after operation, the effectiveness rate was 94.0 %. The recurrence rate was lower in the research, because the local inflammation and scarring formation in simultaneous operation were relatively more severe and consequently the adhesion was firmer. And this also indicated that fibrin glue was not suitable for scleral fixation in our research. Because the application of fibrin glue tended to make less inflammatory than the application of suturing [17].

This research adopted the method of pterygium excision and conjunctival autotransplantation combined with sclera fixation followed by therapeutic contact lens for simultaneous treatment of pterygium complicated with conjunctivochalasis. The redundant conjunctival tissues with rich limbal stem cells were used fully in the simultaneous surgery [18]. The significances of simultaneous treatment were concluded as follows. (1) It not only solved the problem of relaxation and accumulation of the underlying conjunctiva but also supplied sufficient limbal stem cells and conjunctival graft to the nasal surgery area. It played roles of promoting the healing of corneal epithelium, inhibiting the growth of fibrous tissue, reconstructing the barrier function of corneal epithelial and reducing the recurrence rate of pterygium. (2) Some researches showed that pterygium and conjunctivochalasis were related to the reduction of tear mucoprotein [19]. And the conjunctival graft with limbal stem cells was transplanted to the exposed area of nasal sclera, which could protect conjunctival tissue for maximum, reduce the removal of conjunctival tissue and preserve the conjunctival goblet cells as far as possible. And it avoided continuing to reduce the tear mucoprotein. (3) It was reported that surgical trauma and postoperative inflammation would lead to activation of residues in tissue of pterygium fibroblasts and activation of vascular cell, and then formation of fibrovascular tissue, thus causing the recurrence of pterygium after surgery. The simultaneous operation with autologous transplantation effectively avoided more inflammation, rejection or other complications brought by allograft or secondary surgery, and could reduce the recurrence of pterygium. (4) Simultaneous surgery was good for ocular surface reconstruction and cosmetic result, effectively improved the appearance and comfort. In addition, simultaneous surgery was easily accepted by patients owing to the shorter treatment period, less cost and other qualities.

## Conclusions

As a result, the authors claimed that pterygium excision and conjunctival autotransplantation combined with sclera fixation followed by therapeutic contact lens was a safe and effective option in simultaneous treatment of pterygium complicated with conjunctivochalasis.
